# Proteasome serves as pivotal regulator in *Angiostrongylus cantonensis*-induced eosinophilic meningoencephalitis

**DOI:** 10.1371/journal.pone.0220503

**Published:** 2019-08-15

**Authors:** An-Chih Chen, Ling-Yuh Shyu, Yi-Chieh Lin, Ke-Min Chen, Shih-Chan Lai

**Affiliations:** 1 Department of Neurology, Chung-Shan Medical University Hospital, Taichung, Taiwan; 2 Department of Parasitology, Chung Shan Medical University, Taichung, Taiwan; University of Illinois at Chicago, UNITED STATES

## Abstract

Proteasome primarily degrades the unneeded or damaged proteins by proteolysis. Disruption of the brain barrier and its resulting meningoencephalitis caused by *Angiostrongylus cantonensis* are important pathological events in non-permissive hosts. In this study, the results showed upregulated proteasome during *A*. *cantonensis* infection. Occludin degradation and matrix metalloproteinase-9 (MMP-9) activity were significantly increased in infected mice than in uninfected mice. Moreover, confocal immunoflourescence microscopy showed that occludin was co-localized with MMP-9. The infected-mice were treated with proteasomal activity inhibitor MG132 by 1.5 and 3.0 mg/kg/day, which resulted in significantly reduced protein levels of phosphorylated IκBα (*P*<0.05) compared with the untreated control. The phosphorylated nuclear factor kappa-light-chain-enhancer of activated B cells (NF-κB) showed similar result. In addition, MMP-9 activity and occludin degradation were reduced because of MG132 treatment. These results suggested that the proteasome in *A*. *cantonensis* infection degraded phosphorylated IκBα, modulated phosphorylated NF-κB, and then regulated the activation of MMP-9 and occludin degradation. Proteasome alterations were presented in eosinophilic meningitis of BALB/c mice and may contribute to the pathophysiology of eosinophilic meningitis by increasing occludin degradation. This molecule would serve as pivotal regulator in *A*. *cantonensis*-induced eosinophilic meningoencephalitis.

## Introduction

The rat nematode lungworm *Angiostrongylus cantonensis* undergoes obligatory intracerebral migration in its hosts and causes cerebral angiostrongyliasis [1]. Severe eosinophilic meningitis [2] or meningoencephalitis [3] may result from infection in non-permissive hosts (e.g., human or mice). Matrix metalloproteinase (MMP)-9 activity is closely associated with angiostrongyliasis meningitis [4, 5]. This enzyme is associated with the disruption of the blood-cerebrospinal fluid (CSF) barrier [6] and blood-brain barrier (BBB) [7] in mice with angiostrongyliasis meningoencephalitis. The degradation of tight junction protein claudin-5 and dysfunction of the blood-CSF barrier in angiostrongyliasis are mediated by MMP-9 via the IκB-α/NF-κB/MMP-9 signaling pathway as described in detail previously [6].

Proteasome degrades ubiquitinated proteins and is the major pathway for intracellular protein degradation [8]. Proteasome-mediated proteolysis is involved in the regulation of several cellular pathways, namely, cell proliferation, apoptosis, inflammatory response, and antigen presentation, by selective degradation of proteins [9, 10]. For instance, proteasome mediates the degradation of the inhibitor of factor kappa B (IκBα) [11], which has an important function in the regulation of inflammation, and has been reported to regulate the degradation of occludin (a tight junction protein) in human embryonic cells [12]. NF-κB transcription factors are critical regulators of many biological processes, such as innate and adaptive immune responses, inflammation, cell proliferation, and programmed cell death [13]. NF-κB activation is under tight regulation by a number of post-translational modifications, including phosphorylation and ubiquitination [14]. IκB serves as the primary inhibitory component of the NF-κB complex. Thus, degradation of IκB leads to the activation of NF-κB, facilitating the translocation of NF-κB to the nucleus where it serves as a key transcription factor [15].

NF-κB is tightly regulated by multiple checks and balances to prevent persistent NF-κB activation that could result in deleterious effects on the host [16]. Dysregulation of NF-κB plays an underlying role in chronic inflammation and plays important functions in the expression of proinflammatoy factors. These proinflammatory factors are associated with the dysfunction of the BBB [17]. Therefore, we used *A*. *cantonensis* induced-BBB dysfunction as a model to investigate whether proteasome plays a role in the regulation of transcription factor, proteolytic enzyme, and tight junction protein. We also determined whether proteasome was a key regulator of eosinophilic meningoencephalitis through this mechanism.

## Materials and methods

### Experimental animals

This study was performed with the approval of the Institutional Animal Care and Use Committee of Chung-Shan Medical University and the institutional guidelines for animal experiments. Five-week-old male mice (weighing 20~25g), BALB/c strain, were purchased from the National Laboratory Animal Center, Taipei, Taiwan. They were maintained at 12 h light/dark cycle photoperiod. Mice were provided with Purina Laboratory chow and water *ad libitum*. The animals were kept in a specific pathogen-free room at the Animal Center, Chung-Shan Medical University (Taichung, Taiwan) for more than one week before the experimental infection [6]. Mice were inspected daily for adequacy of food, water, bedding and health conditions. After *A*. *cantonensis* infection, mice were monitored for signs of illness (ruffled fur, decreased activity, or tachypnea) and weight loss. No mortality in mice was observed during the period of infection and drug treatment. Mice were maintained under CO2 flow for at least one minute after respiratory arrest. Cervical dislocation was performed as a confirmatory euthanasia method prior to necropsy.

### Reagents

Anti-mouse monoclonal antibodies IκB-α, p-IκB-α, NF-κB, and p-NF-κB generated in rabbits were purchased from the Cell Signaling Technology (Beverly, MA). Rabbit anti-mouse MMP-9 polyclonal antibody was purchased from Abcam (Cambridge, UK). Goat anti-mouse MMP-9 polyclonal antibody was purchased from R&D systems (Minneapolis, MN, USA). Goat anti-mouse proteasome β5 polyclonal antibody and goat anti-mouse occludin polyclonal antibody were purchased from Santa Cruz Biotechnology (CA, USA). Mouse anti-mouse β-actin monoclonal antibody was purchased from Sigma (St. Louis, MO, USA). Horseradish peroxidase (HRP)-conjugated anti-rabbit IgG, HRP-conjugated anti-goat IgG, HRP-conjugated anti-mouse IgG, Rhodamine Red X (RRX)-conjugated anti-rabbit IgG, and DyLight 488-conjugated anti-goat IgG were purchased from Jackson ImmunoResearch Laboratories (West Grove, PA, USA).

### Larval preparation

Third-stage (infective) larvae of *A*. *cantonensis* were obtained originally from wild giant African snails (*Achatina fulica*) propagated for several months and infected with *A*. *cantonensis* L1 by rats (the definitive host) at the Wufeng Experimental Farm (Taichung, Taiwan). The L3 within tissues were recovered using the method of Parsons and Grieve [18] but with some modifications. Briefly, snail shells were crushed, and the tissues were homogenized in a pepsin-HCl solution (pH 1–2, 500 IU pepsin/g tissue) and digested with agitation at 37°C for 2 h. The larvae in the sediment were collected by serial washes in double-distilled water and counted under a microscope. The identity of the L3 larvae of *A*. *cantonensis* was confirmed as described [19]. The third-stage larvae ranged from 425 to 524 μm in length, and 23 to 34 μm in width. The posterior end of the tail always terminates as a fine point. To determine if the larvae found were *A*. *cantonensis*, we have been to feed larvae to rats and then examine their brains 2 to 3 weeks later for evidence of infection.

### Animal infection

A total of 120 male mice were randomly allocated to six groups of 20 mice each. Mice were prohibited food and water for 12 h before infection. The experimental groups (D_5_, D_10_, D_15_, D_20_, and D_25_) were infected with 50 *A*. *cantonensis* larvae by oral inoculation and sacrificed on days 5, 10, 15, 20, or 25 post-inoculation (PI), respectively. The control mice received only water and were sacrificed on day 25 PI. The brains were rapidly removed and frozen in liquid nitrogen. The mice were kept in our laboratory for more than one week prior to experimental infection.

### Treatment of animals

A total of 20 mice were randomly divided into two treated groups (10 mice/group). Two groups of MG132-treated mice were infected with 50 larvae and treated with 1.5 or 3.0 mg/kg/day MG132 (Cayman Chemical, Ann Arbor, MI) for 20 consecutive days. A group of 10 mice, 5 mice for biochemical analysis (Western blotting and Zymography) and 5 mice for Evans blue analysis. Mice were sacrificed at 22 days after inoculation; the brains and CSF were excised and collected for biochemical analysis.

### CSF collection

Mice were anesthetized by intraperitoneal urethane (1.25 g/kg) injection. The mouse was placed in a stationary instrument at 135° from the head and body. The skin of the neck was shaved and swabbed thrice with 70% ethanol. The subcutaneous tissue and muscles were separated. Capillary tube was inserted through the dura mater into the citerna magna and CSF was poured into the capillary tube as described in detail previously [6]. The CSF was injected into a 0.5 mL Eppendorf tube and centrifuged at 3000×g at 4°C for 5 min. The supernatant was collected in a 0.5 mL Eppendorf tube and kept in a freezer at −80°C.

### Western blot analysis

The expression of proteins was evaluated by Western blot analysis. The brain homogenates were centrifuged at 10,000 ×g at 4°C for 10 min to remove the debris. Then, the protein contents (30 μg) of supernatant were determined with protein assay kits (Bio-Rad, Hercules, CA, USA) using bovine serum albumin (BSA) (Sigma-Aldrich Corporation, St. Louis, MO, USA) as the standard. The protein contents were diluted at 1:1 in the loading buffer (2% glycerol, 10% SDS, 2-mercaptoethanol, 5% bromophenol blue, and 0.5 M Tris-HCl; pH 6.8). The mixture was boiled for 5 min before the samples were subjected to SDS-PAGE at room temperature and 110 V for 90 min and electrotransferred to polyvinylidene fluoride (PVDF) membranes (Pall Corporation, Coral Gables, FL, USA) at a constant current of 30 V at 4°C overnight. Afterward, the PVDF membranes were washed twice in PBS containing 0.1% Tween 20 (PBS-T) for 10 min at room temperature. This process was performed thrice. The PVDF membranes were blocked with 5% non-fat dry milk in PBS at 37°C for 1 h and saturated thrice with PBS-T for 10 min at room temperature. The PVDF membranes were reacted with primary antibodies diluted at 1:1000 at 37°C for 1 h. After three washes with PBS-T, the PVDF membranes were incubated with the HRP-conjugated secondary antibodies diluted at 1:10000 at 37°C for 1 h to detect the bound primary antibody. The labeled proteins were visualized by enhanced chemiluminescence (Amersham Biosciences, Amersham, UK), and the densities of the specific immunoreactive bands were quantified with the computer-assisted imaging densitometer system used in the gelatin zymography.

### Confocal scanning laser microscopy

The localization of occludin and MMP-9 were investigated by confocal laser scanning immunofluorescence microscopy. Brain tissues were processed with immunocytochemistry until reaching the primary antibody step. Subsequently, slides were incubated in a mixture of primary antibodies, anti-mouse occludin polyclonal antibody/anti-mouse MMP-9 polyclonal antibody, diluted 1:100 at 37°C for 3 h. After rinsing three times in PBS, using secondary antibodies [Dylight 488-conjugated donkey anti-mouse IgG (H+L) Ab and rhodamine red-conjugated donkey anti-goat IgG (H+L) Ab] the slides were diluted 1:200 at 37°C for 1 h. The nuclei were counterstained with 4'-6-Diamidino-2-phenylindole (DAPI) followed by a wash with PBS. The slides were then mounted with 50% glycerol in PBS. After mounting, the slides were preserved at 4°C until examined under a Zeiss LSM 510 META confocal microscope (Heidelberg, Germany).

### Co-immunoprecipitation

To prevent non-specific adsorption, protein A/G agarose beads (Invitrogen Corporation, Carlsbad, CA, USA) were washed four times with PBS and then incubated with 5% BSA at 4°C for 1 h. Briefly, the solution was centrifuged twice at 10,000 g at 4°C for 2 min to remove supernatant before use. The occludin antibodies were then incubated with sample (1 mg) at 4°C overnight and collected by binding to protein A/G agarose beads. The beads were then washed twice in dissociation buffer (0.5 m tris-HCl, pH 8.0, 120 mM NaCl, 0.5% Triton X-100). Bound proteins were resolved by SDS-PAGE and the target protein MMP-9 association was determined by immunoblotting.

### Gelatin zymography

Activity of gelatinolytic enzymes were determined by gelatin zymography as previously described [20]. The brain homogenates were centrifuged at 10,000 ×g at 4°C for 10 min to remove the debris. The resultant samples (30 μg) were added to an equal volume of standard loading buffer and not boiled before loading. The samples were loaded on 7.5% (mass/volume) in polyacrylamide gels copolymerized with 0.1% gelatin for SDS-polyacrylamide gel electrophoresis (PAGE) (Sigma-Aldrich Corporation, St. Louis, MO, USA) to measure the gelatinase activities. SDS-PAGE was performed in running buffer (25 mM Tris, 250 mM glycine, 1% SDS) at room temperature and 110 V for 1 h. After electrophoresis, each gel was washed twice for 30 min at each instance in denaturing buffer (2.5% Triton X-100) at room temperature and washed twice with double-distilled water at room temperature for 10 min. Then, the gel was incubated in the reaction buffer (50 mM Tris-HCl, pH 7.5; containing 10 mM CaCl2, 0.02% Brij-35, and 0.01% NaN3) at 37°C for 18 h. The gel was stained with 0.25% Coomassie Brilliant Blue R-250 (Bio-Rad, Hercules, CA, USA) for 1 h and destained in 15% methanol/7.5% acetic acid. The final gel had a uniform background except in regions where the gelatinolytic enzymes had migrated and cleaved their respective substrates. The gelatinolytic enzyme was quantitatively analyzed using a computer-assisted imaging densitometer system (UN-SCAN-ITTM gel Version 5.1, Silk Scientific, Provo, Utah, USA).

### BBB permeability evaluation

BBB permeability was assessed using Evans Blue concentrations in the brains. The mice were injected with 2% (w/v) Evans blue dye (5 mL/kg body weight; Sigma, St. Louis, MO, USA) in saline via the tail vein. After 2 h of circulation, the mice were anesthetized and transcardially perfused with saline to remove intravascular dye. The brains were weighed and homogenized in a 50% trichloroacetic acid solution. The homogenates were centrifuged at 12,000 ×g for 10 min, and the supernatants were collected. Each supernatant was measured at 620 nm for absorbance to calculate Evans Blue concentrations by using a spectrophotometer (Hitachi U3000, Tokyo, Japan).

### Statistical analysis

Results in the different groups of mice were compared using the nonparametric Kruskal-Wallis test followed by post-testing using Dunn’s multiple comparison of means. All results were presented as means±standard deviation. Statistical significance was established for *P* values <0.05.

## Results

### Kinetic studies for the proteasome in the mouse brain and CSF

**Fig 1:**
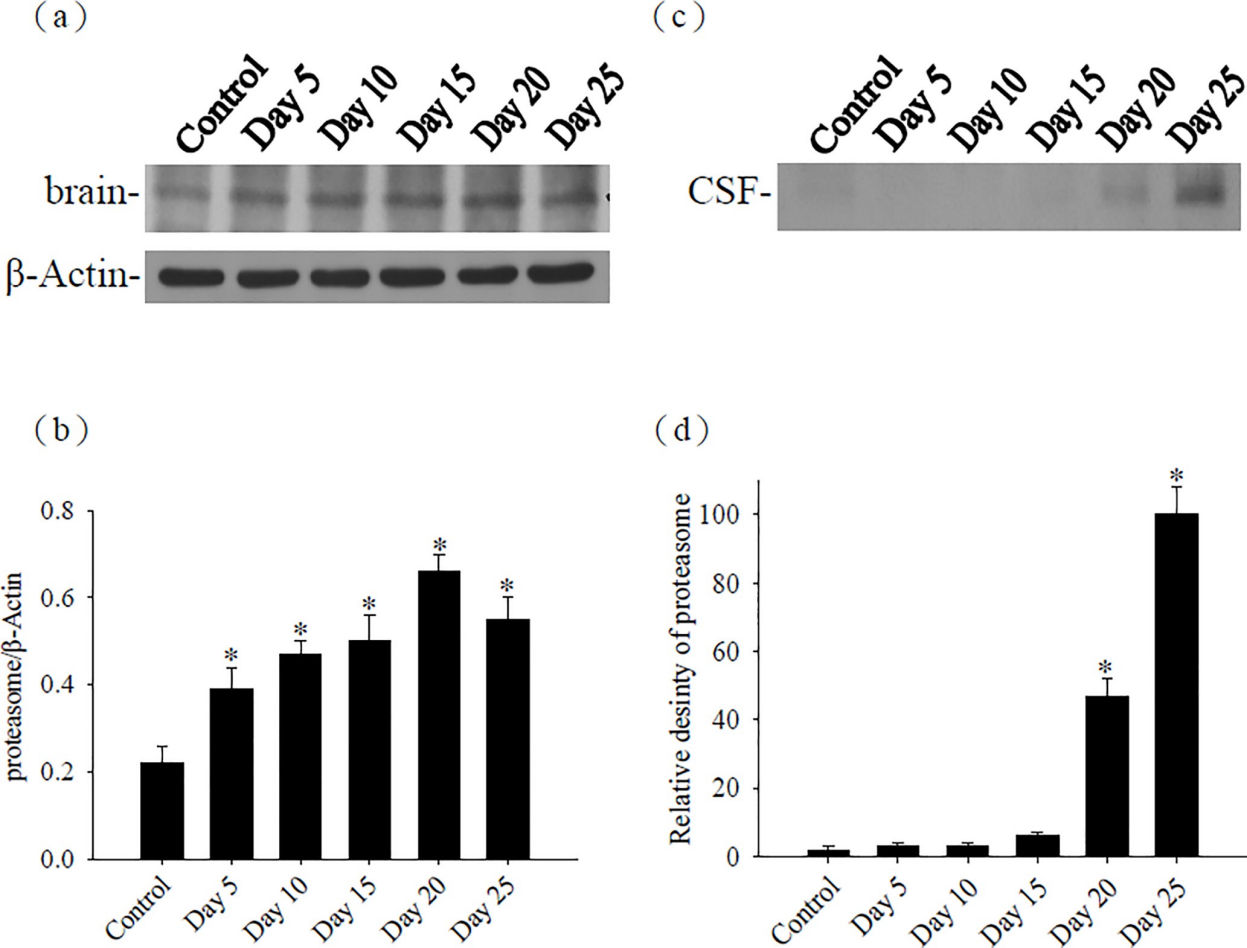
Time-course studies for proteasome. The protein levels of proteasome from the (a) brain and (c) cerebrospinal fluid (CSF) of mice infected with *Angiostrongylus cantonensis*. β-actin was used as a loading control. (b, d) Quantification and normalization of the proteasome to β-actin was performed with a computer-assisted imaging densitometer system. The data are presented as the mean±SD of three independent experiments in duplicate. * indicates a statistically significant difference.

The proteasome in the brains and CSF was determined by Western blot. The proteasome were significantly increased (*P* < 0.05) in the brains of the infected mice on days 5, 10, 15, 20, and 25 than in the uninfected mice. Also, the proteasome in the CSF was significantly increased (*P*<0.05) on days 20 and 25 PI in the infected mice than in the uninfected mice (Fig 1).

### Kinetic studies for occludin in the mouse brain and CSF

**Fig 2:**
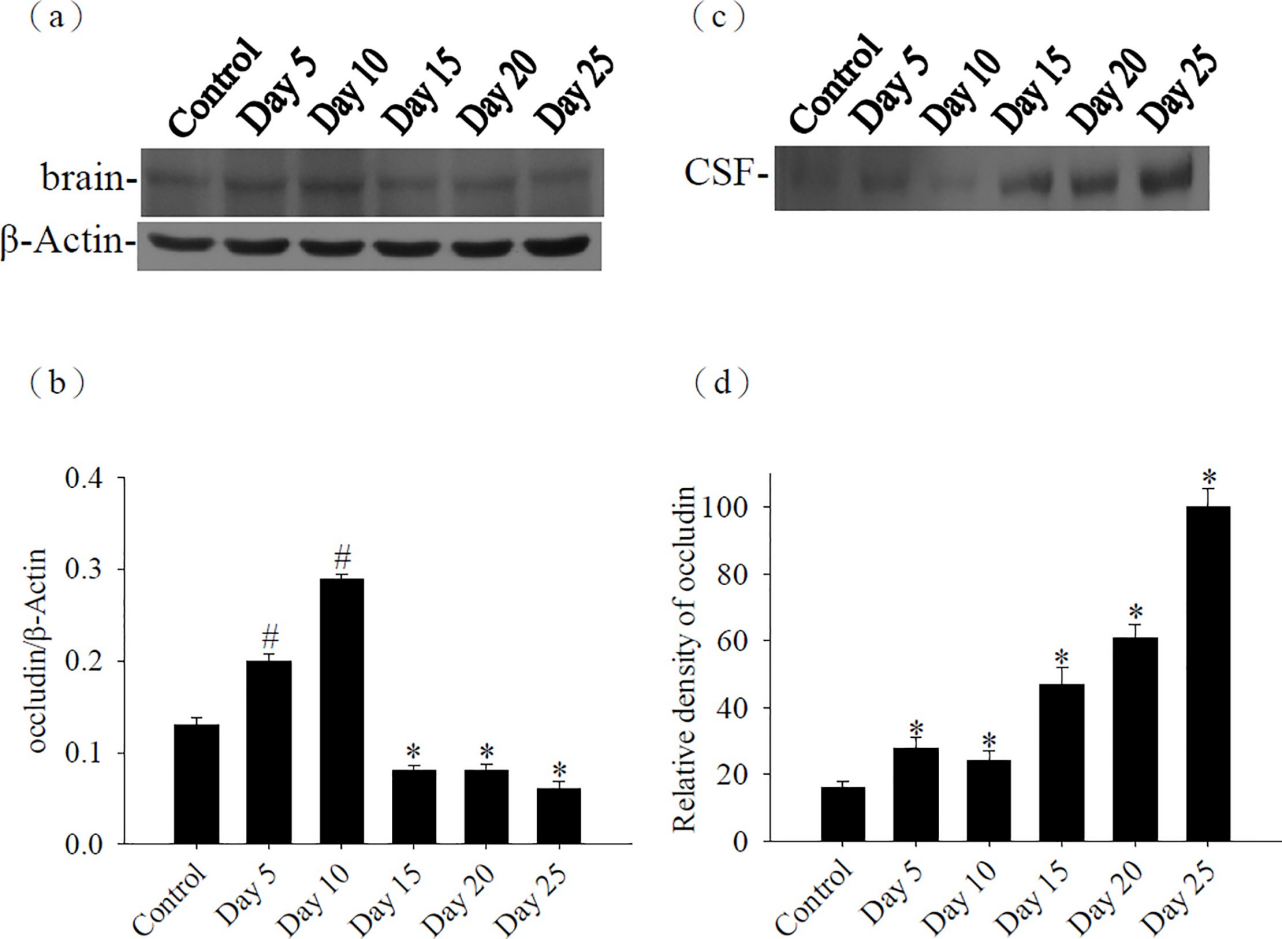
Time-course studies for occludin. (a) The protein levels of occludin from the brain of mice infected with *Angiostrongylus cantonensis*. β-actin was used as a loading control. (b) Quantification and normalization of occludin to β-actin was performed with a computer-assisted imaging densitometer system. ^#^ indicates a statistically significant increase. * indicates a statistically significant decrease. (c) The protein levels of occludin from the CSF of mice infected with *A*. *cantonensis*. (d) The relative density was quantified using a computer-assisted imaging densitometer system. * indicates a statistically significant increase.

The occludin in the brains and CSF was also detected by Western blot. The occludin in the brains was significantly increased (*P*<0.05) on days 5 and 10 PI in the infected mice than in the uninfected mice. However, occludin on days 15, 20, and 25 PI were significantly decreased. Occludin in the CSF was significantly increased (*P*<0.05) on days 5, 10, 15, 20, and 25 PI in the infected mice than in the uninfected mice (Fig 2).

### Co-localization of occludin and MMP-9 in the epithelial cells of the mouse choroid plexus on day 20 PI

**Fig 3:**
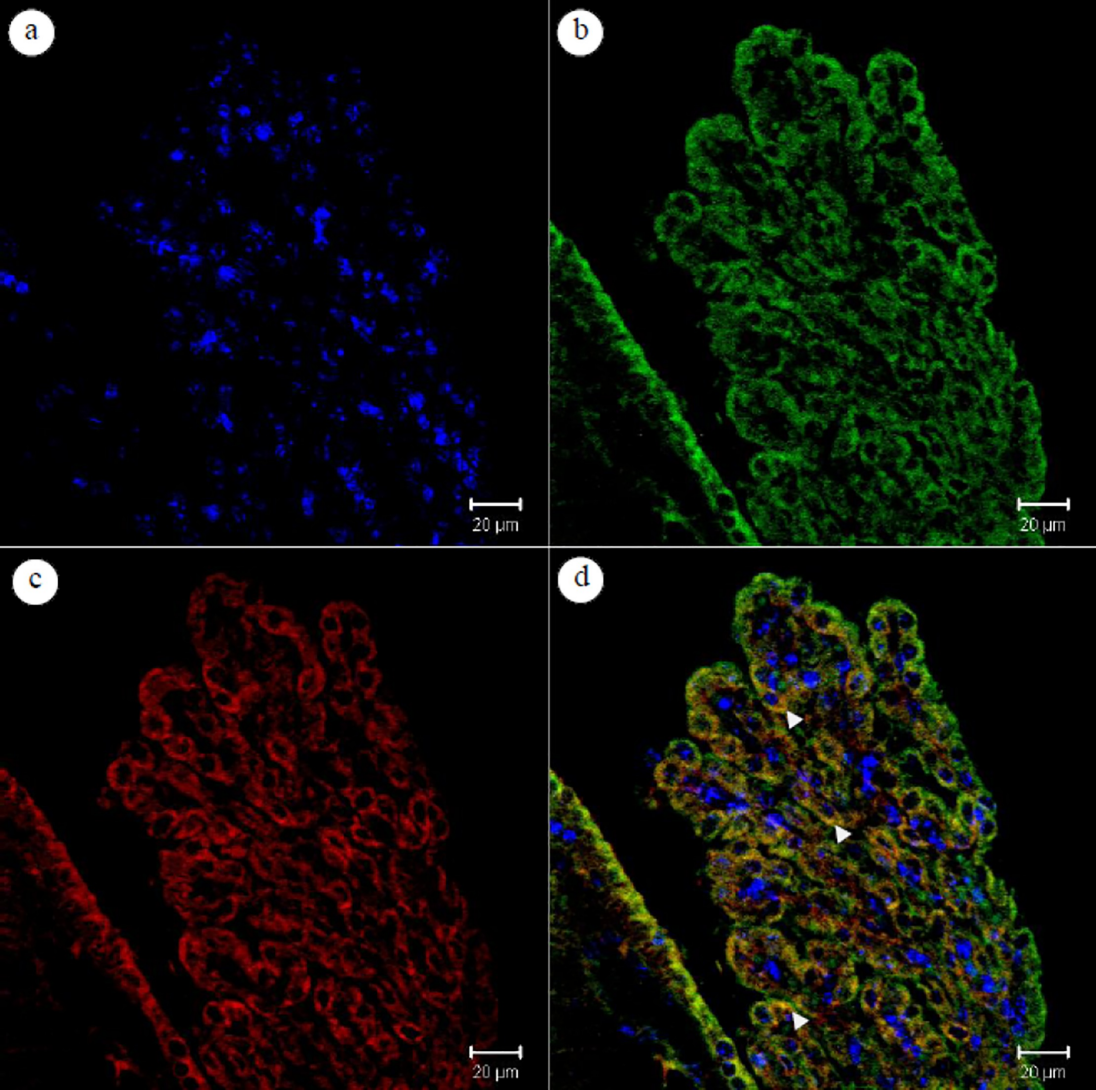
Localization of occludin and matrix metalloproteinase (MMP)-9 in the choroid plexus of the mouse brain. (a) The epithelium of the choroid plexus were stained with 4’,6-diamino-2-phenylindole (DAPI) for nuclei (blue); (b) DyLight 488-stained for occludin (green); (c) Rhodamine red-stained for MMP-9 (red); (d) The merged image shows colocalization of Dylight 488-labeled peptide H4 with Rhodamine B-labeled lipid in the epithelial cells of choroid plexus (yellow, d). Scale bar = 20 μm.

Confocal laser scanning immunofluorescence microscopy measured the co-localization of occludin and MMP-9 in *A*. *cantonensis*-infected mouse brain sections. Occludin was stained around the epithelium of the choroid plexus, and MMP-9 was localized in the epithelial cells. Merged images showed the expression of MMP-9 and occludin at the same sites around the epithelium in the choroid plexus after infection with *A*. *cantonensis* (Fig 3).

### Interaction between occludin and MMP-9

**Fig 4:**
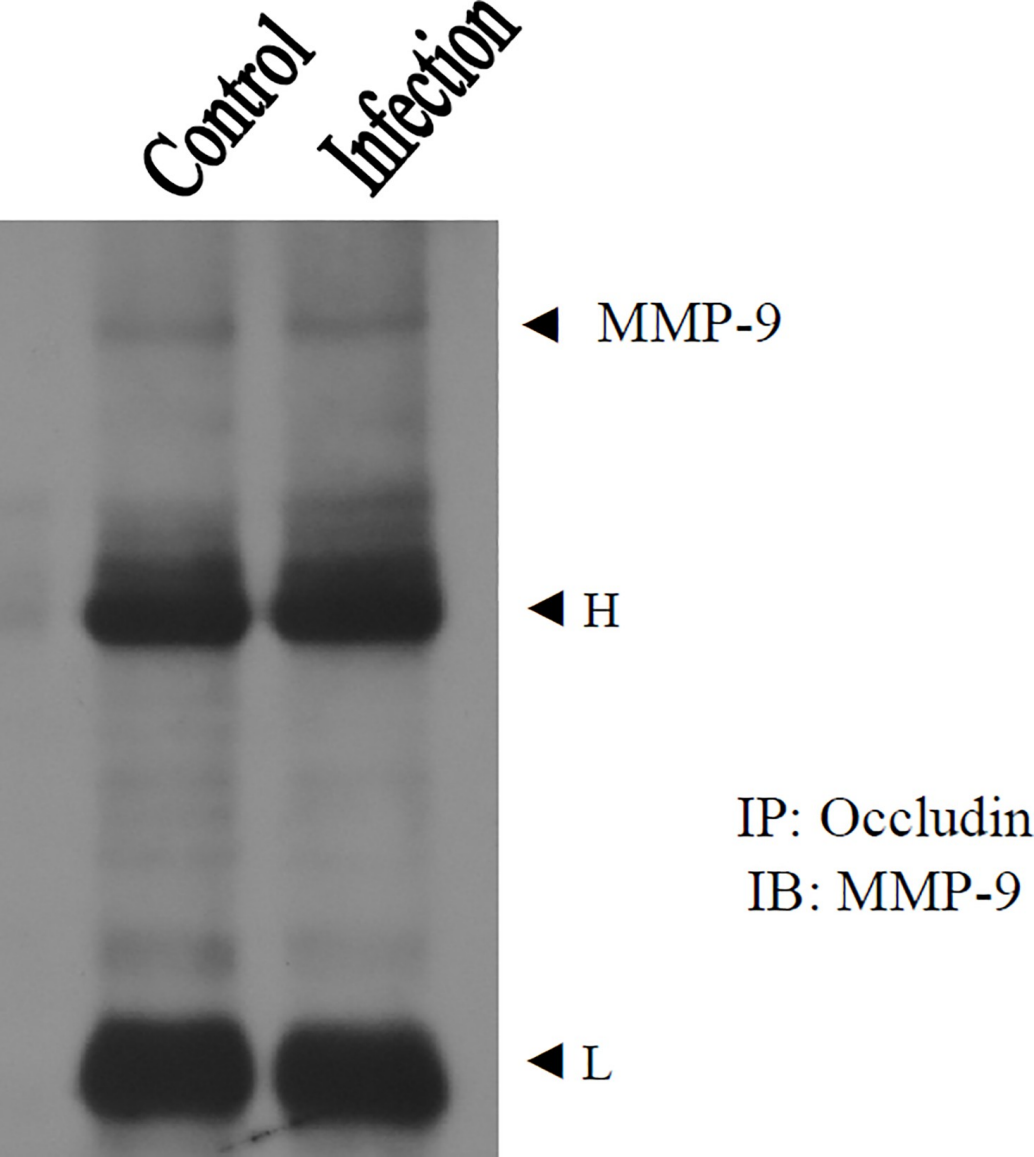
Interaction between occludin and MMP-9 in mice infected with *Angiostrongylus cantonensis*. Occludin interacted with MMP-9 in brain homogenates. Control, uninfected mice. Infection, mice infected with *A*. *cantonensis* on day 20 post-infection. The arrowheads indicate MMP-9 bands. H, immunoglobulin heavy chain. L, immunoglobulin light chain. Immunoprecipitates (IP): occludin. Immunoblotting (IB): MMP-9.

Co-immunoprecipitate is a powerful technique that is used regularly by molecular biologists to analyze protein-protein interactions that uses an antibody to immunoprecipitate the antigen (bait proteins) and co-immunoprecipitate any interacting proteins (prey proteins). While detected the prey proteins exist by immunoblotting, confirming bait proteins and prey proteins were interaction. Western blot analysis showed occludin in mice infected with *A*. *cantonensis* on day 20 PI. Thus, we used this time point to investigate whether occludin interacted with MMP-9. Brain homogenates were subjected to immunoprecipitation with anti-mouse occludin antibody followed by anti-mouse MMP-9 immunoblotting. Results showed that occludin interacted with MMP-9 (Fig 4).

### Influence of IκB-α after treatment with MG132

**Fig 5:**
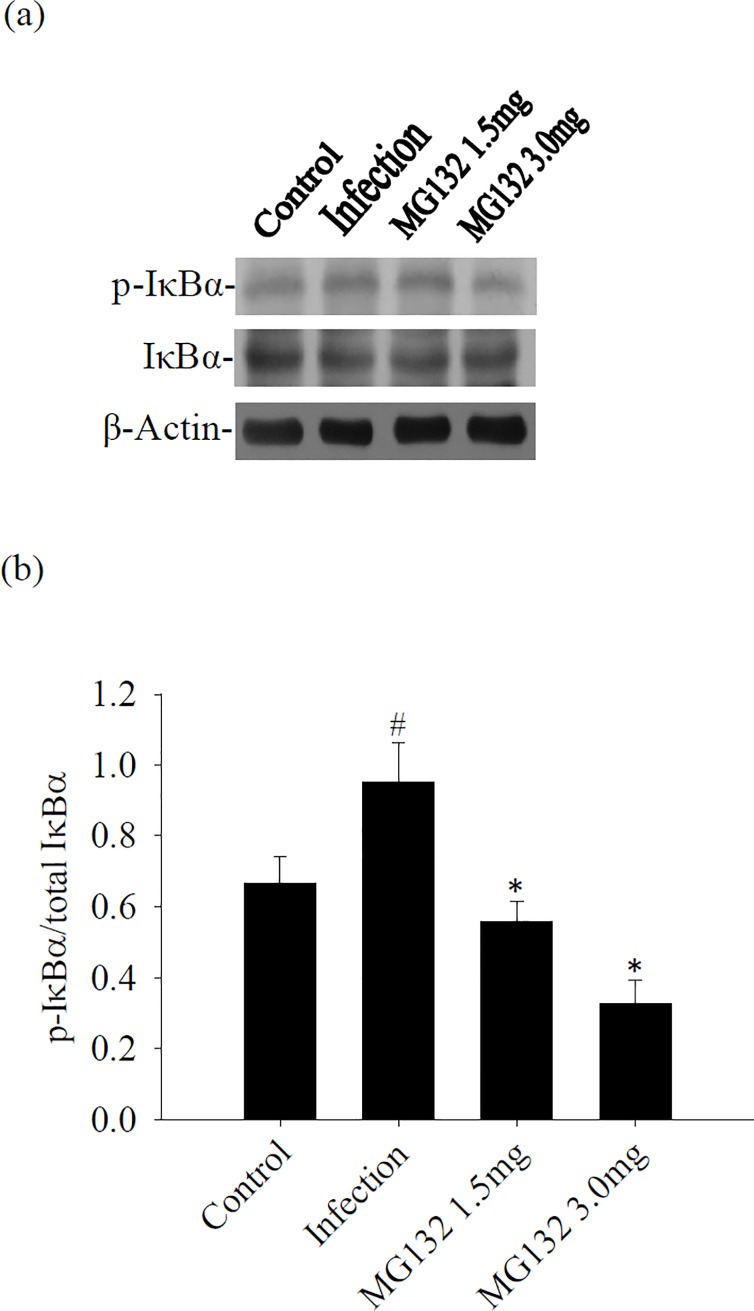
Influence of treatment on IκB-α and p-IκB-α in the brain. (a) Protein bands of IκB-α and p-IκB-α from the brain of mice infected with *A*. *cantonensis* and treated with MG132. β-actin was used as a loading control. (b) Quantification of phosphorylation ratios [intensity of phosphorylated IκB-α (p-IκB-α) divided by the intensity of total IκB-α (t-IκB-α)] following normalization for β-actin. The data are presented as the mean± SD of three independent experiments in duplicate. ^#^ indicates a statistically significant increase.* indicates a statistically a significant decrease.

The effect of MG132 on IκB-α and pIκB-α was investigated in a murine angiostrongyliasis model. Western blot results showed that pIκB-α/IκB-α increased significantly in the brain of infected mice than in the uninfected controls. By contrast, pIκB-α/IκB-α was significantly reduced after treatment with MG132 (Fig 5).

### Influence of NF-κB after treatment with MG132

**Fig 6:**
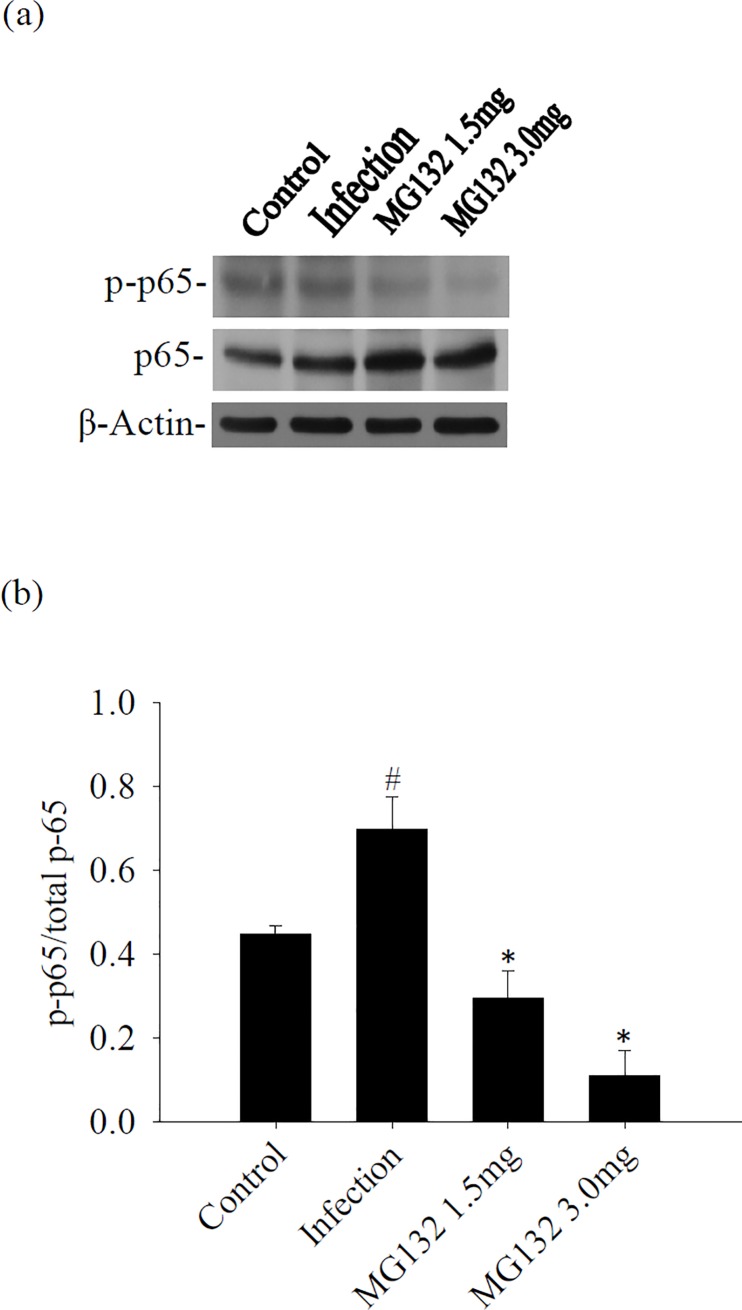
Influence of treatment on NF-κB in the brain. (a) Protein bands of NF-κB p65 and p-p65 from the brain of mice infected with *A*. *cantonensis* and treated with MG132. β-actin was used as a loading control. (b) Quantification of phosphorylation ratios [intensity of phosphorylated p65 (p-p65) divided by the intensity of total p65 (t-p65)] following normalization for β-actin. The data are presented as the mean±SD of three independent experiments in duplicate. ^#^ indicates a statistically significant increase. * indicates a statistically significant decrease.

To determine whether NF-κB could be responsible for the proteasome, we measured the levels of NF-κB p65 and p-p65 in a murine angiostrongyliasis model. Western blot results showed that p-p65/p65 was significantly increased in the brain of infected mice than in the uninfected controls. However, p-p65/p65 was significantly reduced after treatment with MG132 (Fig 6).

### Influence of MMP-9 after treatment with MG132

**Fig 7:**
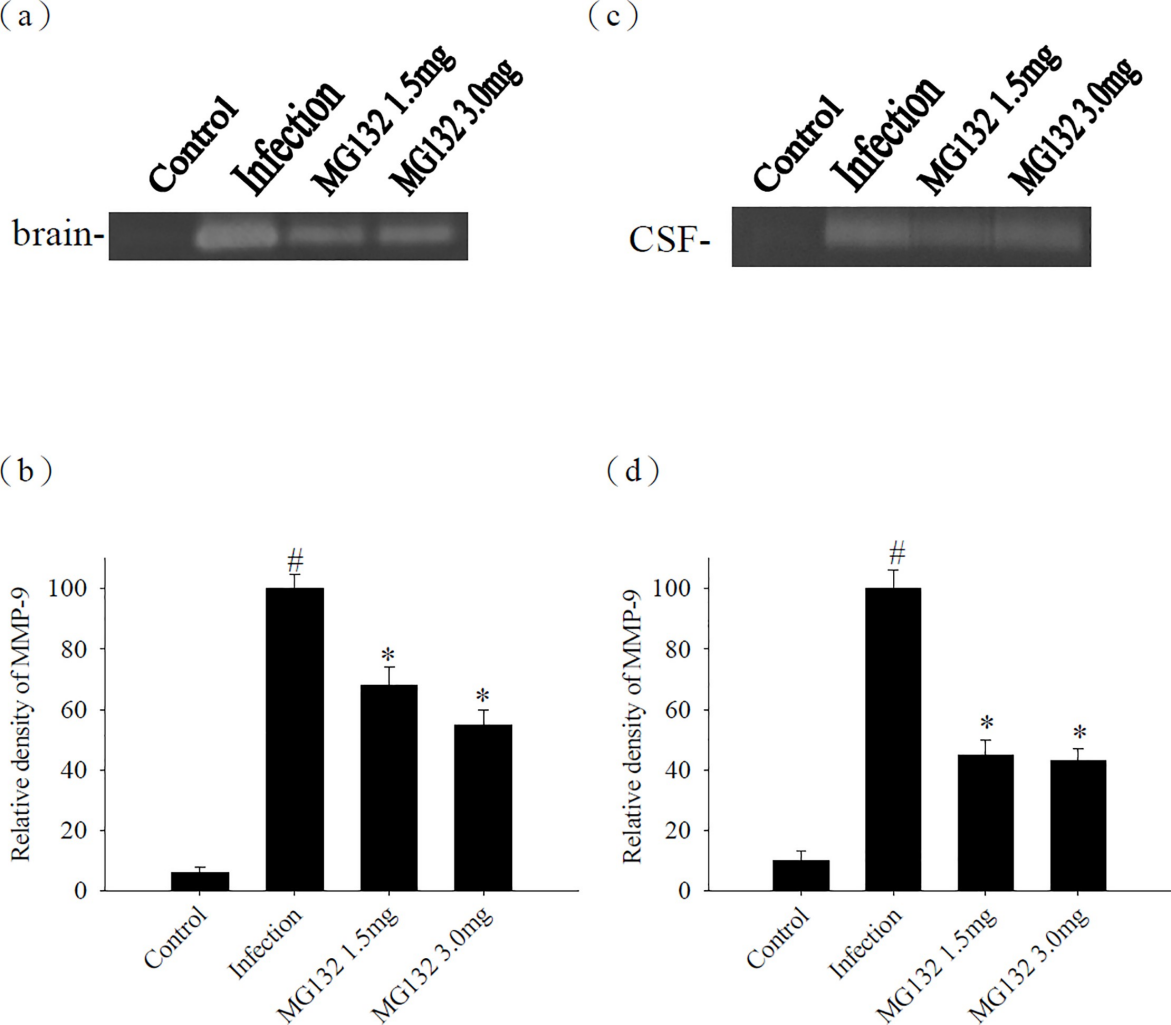
Influence of treatment on MMP-9. The activities of MMP-9 from the (a) brain and (c) CSF of mice infected with *A*. *cantonensis* and treated with MG132. (b, d) The relative intensity was quantified using a computer-assisted imaging densitometer system. Bars represent mean ± S.D. (n = 5 per group). ^#^ indicates a statistically significant increase.* indicates a statistically significant decrease.

We investigated whether MMP-9 expression could be influenced by the proteasome in a murine angiostrongyliasis model. We assessed the activity of MMP-9 by zymography and found that MMP-9 activity significantly decreased after treatment with MG132 compared with that in the untreated-infected controls (Fig 7).

### Influence of occludin after treatment with MG132

**Fig 8:**
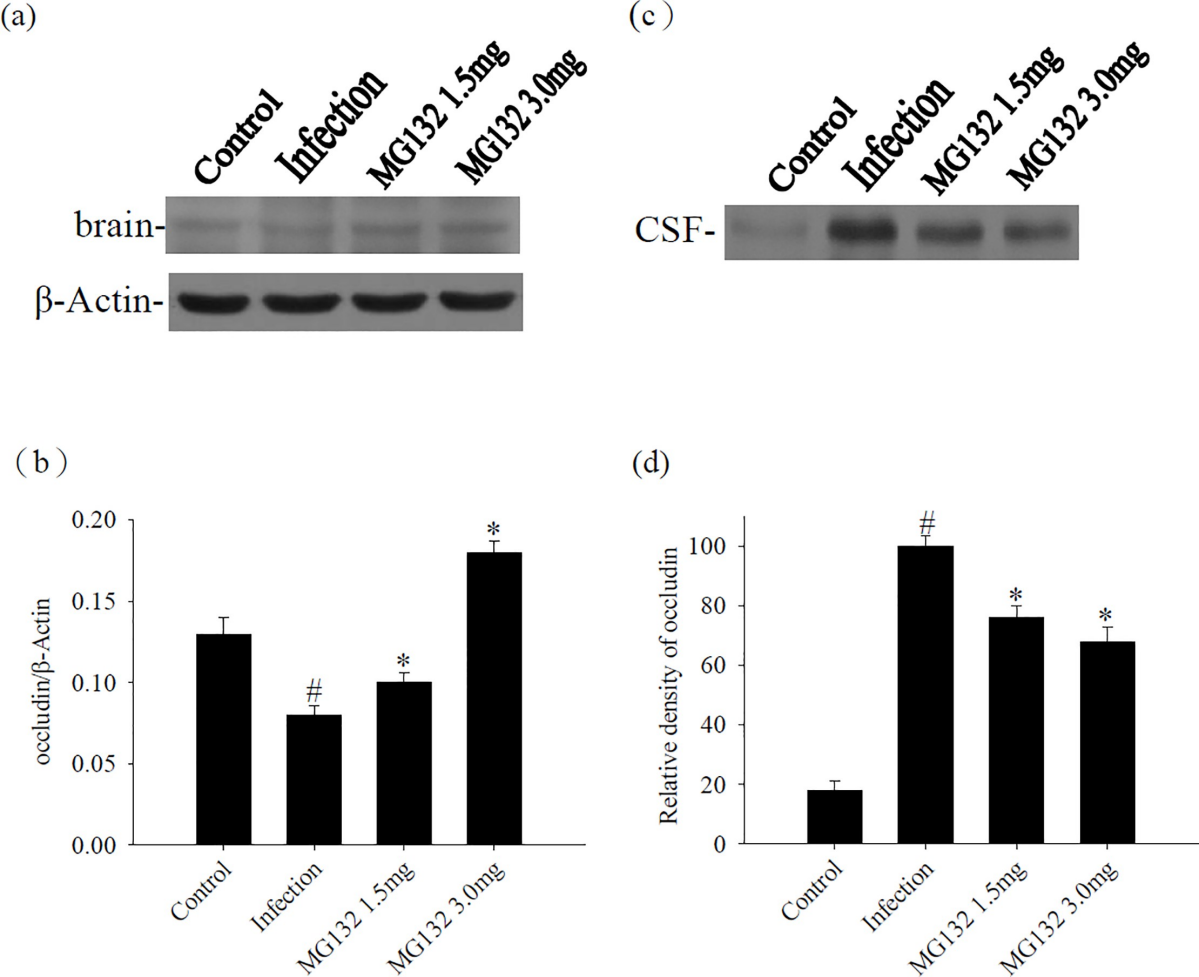
Influence of treatment on occludin. (a) The protein levels of occludin from the brain of mice infected with *Angiostrongylus cantonensis* and MG132 treatment. β-actin was used as a loading control. (b) Quantification and normalization of occludin to β-actin were performed with a computer-assisted imaging densitometer system. ^#^ indicates a statistically significant decrease. * indicates a statistically significant increase. (c) The protein levels of occludin from the CSF of mice infected with *A*. *cantonensis* and MG132 treatment. (d) The relative density was quantified using a computer-assisted imaging densitometer system. ^#^ indicates a statistically significant increase. * indicates a statistically significant decrease. Bars represent mean ± S.D. (n = 5 per group).

We elucidated the putative role of the proteasome in the tight junction protein degradation in a murine angiostrongyliasis model. We focused on occludin expression, which was downregulated significantly in the brain but upregulated significantly in the CSF of the infected mice compared with the uninfected controls. The occludin was significantly upregulated in the brain after treatment with MG132. By contrast, occludin was significantly downregulated in the CSF upon treatment by MG132 (Fig 8).

### Influence of BBB permeability after MG132 treatment

**Fig 9:**
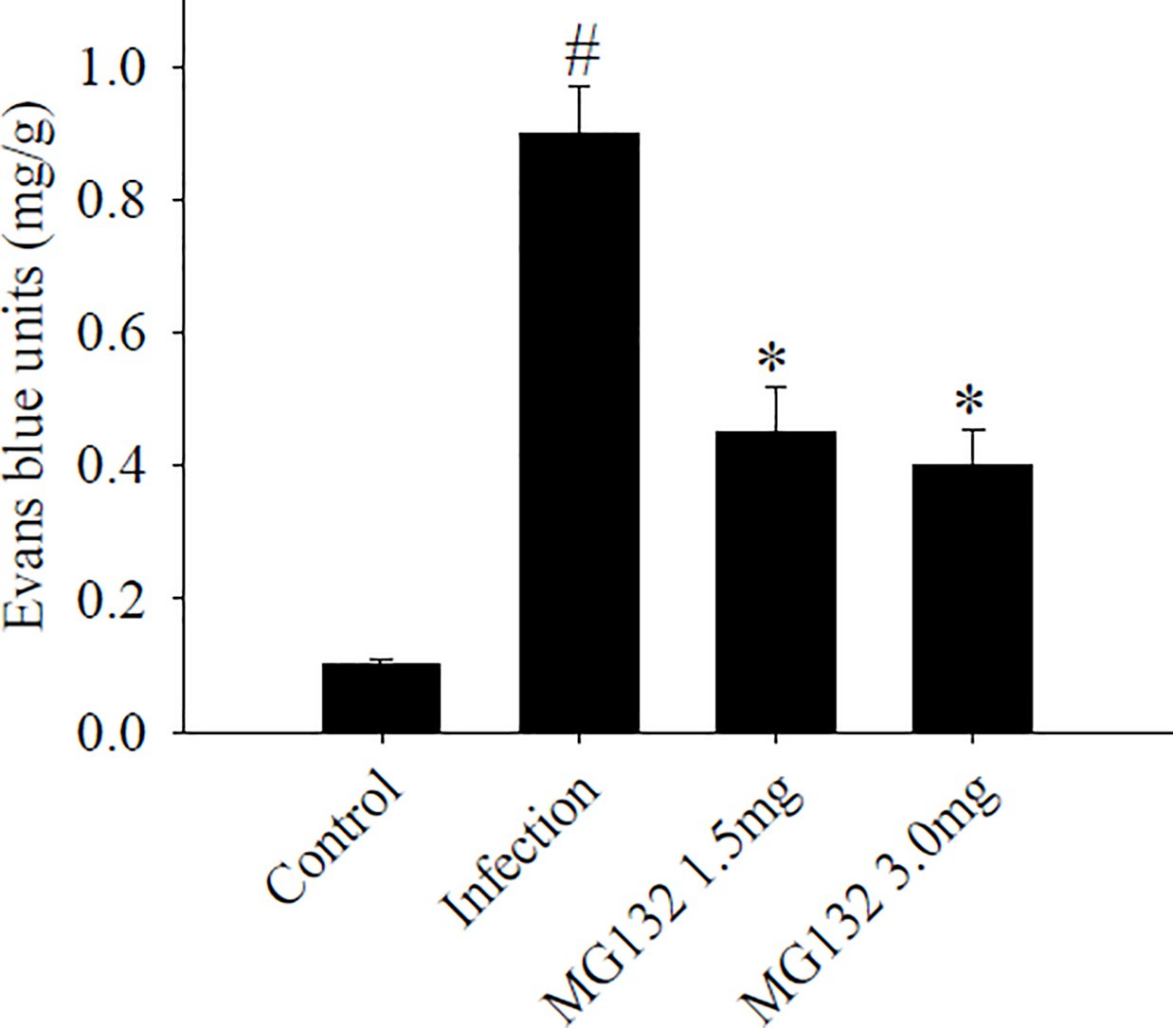
Influence of MG132 on BBB permeability. Mouse BBB permeability was detected by performing Evans Blue extravasation during *Angiostrongylus cantonensis* infection. The Evans blue units were significantly decreased (*P* < 0.05) in the MG132 treatment group compared with those of the *A*. *cantonensis*-infected mice. ^#^ indicates a statistically significant increase. * indicates a statistically significant decrease.

Evans Blue concentration can be an indicator of mouse BBB leakage during *A*. *cantonensis* infection and after MG132 treatment. BBB permeability was significantly increased in mice infected with *A*. *cantonensis* compared with controls. Following treatment, BBB permeability was significantly decreased in the MG132 treatment group compared with the infected group (Fig 9).

## Discussion

The giant size (660–700 kD) of the proteasome conflicts with its passage through the basal lamina of the intact BBB via diffusion [21]. Previous studies assumed that this characteristic represents the transportation mechanism of proteasomes into the CSF [22]. Additionally, other routes of secretion, such as exosomes, plasma membrane-derived exovesicles, and caveolae-derived transport across the choroid plexus epithelium, have to be considered [22, 23, 24]. Our results demonstrated that the proteasome was presented in the CSF and correlated to eosinophilic meningoencephalitis. These data suggested that the proteasome was secreted into the CSF by the plexus choroideus.

Inflammation involves the sequential activation of signaling pathways leading to the production of both pro- and anti-inflammatory mediators. NF-κB is inferred to have an important role in the induction of pro-inflammatory gene expression and angiostrongyliasis meningitis [6, 25]. Histone deacetylase sirtuin-1 is an important transcriptional regulatory protein attributed to the inhibition of NF-κB and reduces eosinophil infiltration in angiostrongyliasis meningoencephalitis [26]. The NF-κB pathway is one of the major signaling pathways involved in the regulation of inflammatory response. Proteasome degradation is implicated in the activation of NF-κB through the specific degradation of IκBα, the inhibitor of NF-κB [27]. In the present study, significant elevation of p-IκB-α and p-NF-κB was demonstrated in the brain samples of mice with eosinophilic meningoencephalitis caused by *A*. *cantonensis*. However, mice treated with MG132 had decreased phosphorylation of p-IκB-α and p-NF-κB in eosinophilic meningoencephalitis. These results showed that proteasome was a regulator for NF-κB activation during *A*. *cantonensis* infection.

The brain barrier permeability is partially regulated by tight junction protein, such as occludin. This protein is degraded by the proteasome after ubiquitination using specific E3 ubiquitin-protein ligase, Itch [12]. Japanese encephalitis virus-infected astrocytes have been found to release biologically active molecules that activated ubiquitin proteasome, degraded zonula occludens-1 and claudin-5, and disrupted the endothelial barrier integrity in cultured brain microvascular endothelial cells [28]. In the current study, occludin degradation increased in mice with eosinophilic meningoencephalitis compared with the controls. Occludin degradation was reduced by a proteasome inhibitor, MG132. Moreover, lower BBB permeability was presented in the MG132-treated mice with *A*. *cantonensis* infections. These data suggested that the increased proteasome-mediated degradation of occludin may occur in eosinophilic meningoencephalitis and may contribute, together with other factors, to increase the brain barrier permeability and inflammation. The factors responsible for the increased occludin proteasome-mediated degradation remain unclear. The proteasome played an important function in the regulation of inflammation. Thus, this molecule may have been involved in the regulation of BBB permeability and contribute to parasitic meningitis. Proteasome inhibition might reduce eosinophil infiltration and thereby improve eosinophilic meningitis.

The BBB permeability is enhanced in mice with eosinophilic meningitis or meningoencephalitis, which may be caused by MMP-9 [6] or MMP-12 [29]. These studies demonstrated that MMP-9 and MMP-12 might be associated with the disruption in the BBB during inflammation. Moreover, MMP-induced endothelial barrier disruption is accompanied by MMP-mediated proteolytic degradation of claudin-5 and ubiquitin proteasome-mediated degradation of zonula occludens-1 [28]. In this study, co-immunoprecipitation results showed that MMP-9 interacted with occludin in the infected mice. The protein levels of occludin were increased after *A*. *cantonensis* infection, however, decreased on days 15, 20, and 25 PI. The time points of occludin degradation were match with MMP-9 induction. Elevated MMP-9 increased BBB permeability and worsened brain inflammation after eosinophilic meningoencephalitis. Thus, occludin, degraded by MMP-9, may be released into CSF after eosinophilic meningoencephalitis. In addition, the degradation of occludin was significantly altered upon treatment with MG132, while *A*. *cantonensis*-induced MMP-9 was reduced by treatment with the proteasome inhibitor. Therefore, we proposed that proteasome may contribute to the pathophysiology of eosinophilic meningitis by increasing occludin degradation.

In summary, the proteasome in *A*. *cantonensis* infection degraded phosphorylated IκBα, modulated phosphorylated NF-κB, and then regulated the MMP-9 production and occludin degradation. The results strongly showed that the proteasome served as a pivotal regulator of *A*. *cantonensis*-induced eosinophilic meningoencephalitis in BALB/c mice. These mechanistic insights could be used for the pathophysiologic evaluation of the brain barrier breakdown and provided the basis for therapeutic strategies for *A*. *cantonensis*-induced tight junction disruption.
